# Social Isolation in Long-Term Care Facilities Related to COVID-19: Effect on Resident Anxiety and Care

**DOI:** 10.1093/geroni/igab046.2207

**Published:** 2021-12-17

**Authors:** Nazmus Sakib, Joseph June, Lindsay Peterson

**Affiliations:** 1 Department of Industrial and Management Systems, University of South Florida, Tampa, Florida, United States; 2 University of South Florida, Tampa, Florida, United States; 3 University of South Florida, University of South Florida, Florida, United States

## Abstract

Loneliness is a common problem in long-term care. It has been associated with a higher risk of depression, aggressive behaviors, and anxiety and may be a risk factor for cognitive decline. Loneliness can exacerbate social isolation. The COVID-19 emergency brought on measures in Florida, beginning in March 2020, to separate nursing home (NH) and assisted living community (ALC) residents from each other and family members to limit virus spread. This study examines results of a survey with Florida NH (N=59) and ALC (N=117) administrators concerning effects of these measures. Scaled (1-5, lowest to highest) data indicate that resident anxiety was higher in NHs (M=3.40) than ALCs (M=3.17). Care disruptions related to limited resident-to-resident contact also were worse in NHs (M=3.74) than in ALCs (M=3.21), while care disruptions related to loss of family support were higher among ALCs (M=3.19) than in NHs (M=2.86). Implications of these findings will be discussed.

